# Lessons Learnt from Interregional and Interagency Collaboration in Polio Outbreak Response in the Horn of Africa

**DOI:** 10.29245/2578-3009/2021/S2.1112

**Published:** 2021-04-12

**Authors:** Samuel Okiror, Brigitte Toure, Bob Davis, Rustum Hydarov, Bal Ram, Joseph Okeibunor, Chidiadi Nwogu

**Affiliations:** 1 WHO Horn of Africa Coordination Office (HOA), Nairobi KENYA; 2 UNICEF Nairobi Kenya; 3 American Red Cross, Nairobi Kenya; 4 CORE Group Regional Office Nairobi; 5 WHO Regional Office for Africa (WHO AFRO), Brazzaville, Congo

**Keywords:** Inter-regional collaboration, Polio virus, Outbreak, Lessons learnt

## Abstract

Following the outbreak of poliovirus in the countries in the Horn of Africa, Somalia, Kenya and Ethiopia, in two WHO regions, an outbreak response involving the WHO Africa and WHO East and Mediterranean Regions and partner agencies like the UNICEF in East and Southern African was developed. This paper documents response to polio virus outbreak in the Horn of Africa and the lessons learnt for the interregional and inter-agency collaboration on the response. This collaboration led to speedy interruption of the outbreak and within a period of one year the total virus load of 217 in 2013 was brought down to mere six. This resulted from collaborative planning and implementation of activities to boost the hitherto low immunity in the countries andimprove surveillance among others. A number of lesson were generated from the process. Some of the lessons is critical role such collaboration plays in ensuring simultaneous immunity boosting, information and resources sharing, among other. Some challenges were equally encountered, chiefly in the appropriation of authorities. In conclusion, however, one is safe to note that the collaboration was very fruitful given the timely interruption of transmission.

## Introduction

Global Polio Eradication Initiative (GPEI) recorded major successes, following the setting up of the initiative and declaration of the completion of polio eradication a programmatic and public health emergency in 2012^1–4^. Enormous human, financial and material resources were mobilized and committed to complete the polio eradication, globally^5^. With these resources there was better implementation of core strategies of polio eradication, increased accountability, and implementation of innovative approaches^2^. Substantial progress was made in the fight against polio in the African Region, with cases of wild poliovirus (WPV) dropping from 912 reported in 2008 in 12 countries to 128 cases in 3 countries by the end of 2012^6^.

However, 2013 opened with new setbacks and new outbreaks of polioviruses. An avalanche of poliovirus hit the Horn of Africa (HOA) between the periods of 2013 and 2014, which began with an outbreak of wild poliovirus type 1 (WPV1) in 2013^7–12^. Six cases were confirmed, four from Somalia (Banadir and Bay region) and two from Kenya (Dadaab in north-eastern Kenya). The first case was confirmed in Somalia on 9 May 2013 and in Kenya on 22 May^13, 14^. By 1 July 2013, 25 cases had been reported from Somalia (primarily from Banadir region) and six from Kenya (Dadaab in north-eastern Kenya) and by 14 Aug, Somalia had the worst outbreak reported globally in a non-endemic country with 105 cases confirmed. At the same period close to 10 cases of wild polio were confirmed in Kenya while six cases of polio were confirmed in Ethiopia. The series of outbreaks in HoA threatened the GPEI mandate for an accelerated push and completion of polio eradication within a set timeframe, which was endorsed Polio Eradication and Endgame Strategic Plan 2013–2018 objective of stopping all polio transmission globally by the end of 2014^15^.

Responding to these outbreaks in the HoA GPEI put up a strong team comprising of a collaboration between the two WHO Regional Office for Africa (AFRO) and Regional Office for Eastern Mediterranean (EMRO) as well as UNICEF Regional Office for East and Southern Africa (ESARO). This inter-regional and interagency collaboration responded to the outbreak of WPV1 with renewed determination to win the war against poliomyelitis in the Region^5, 11^. This paper discusses the lessons learnt from that seemingly complex marriage ofpurpose across regions and agencies, necessitated by the scourge of poliomyelitis in the HoA region.

## Inter-Regional and Inter-Agency Collaboration to Combat Polio Outbreaks in The HoA

**The Horn of Africa** is the easternmost extension of African land, often defined as home to the countries of Djibouti, Eritrea, Ethiopia, and Somalia, whose cultures have been linked throughout their long history. Some more restrictive definitions of HoA have the constituting countries as Djibouti, Eritrea, and Ethiopia. While other more elaborate and more common definition include all the countries mentioned above, as well as parts or all of Kenya, Sudan, South Sudan, Uganda, Yemen. Part of the Horn of Africa region is also known as the Somali peninsula, a term typically used when referring to lands of Somalia and eastern Ethiopia.

The HoA is such a diverse area in landmass but in most part culturally similar. However, under the regional arrangements of the World Health Organization, the HoA falls into two separate regions, namely the African and the Eastern Mediterranean regions. Djibouti, Somalia, Sudan are in the Eastern and Mediterranean Region, despite their cultural linkage to Eritrea, Ethiopia and Kenya, which are within the WHO African Region. Somalis populate the eastern part of Kenya and also the Somali region of Ethiopia.

**Figure 1:**
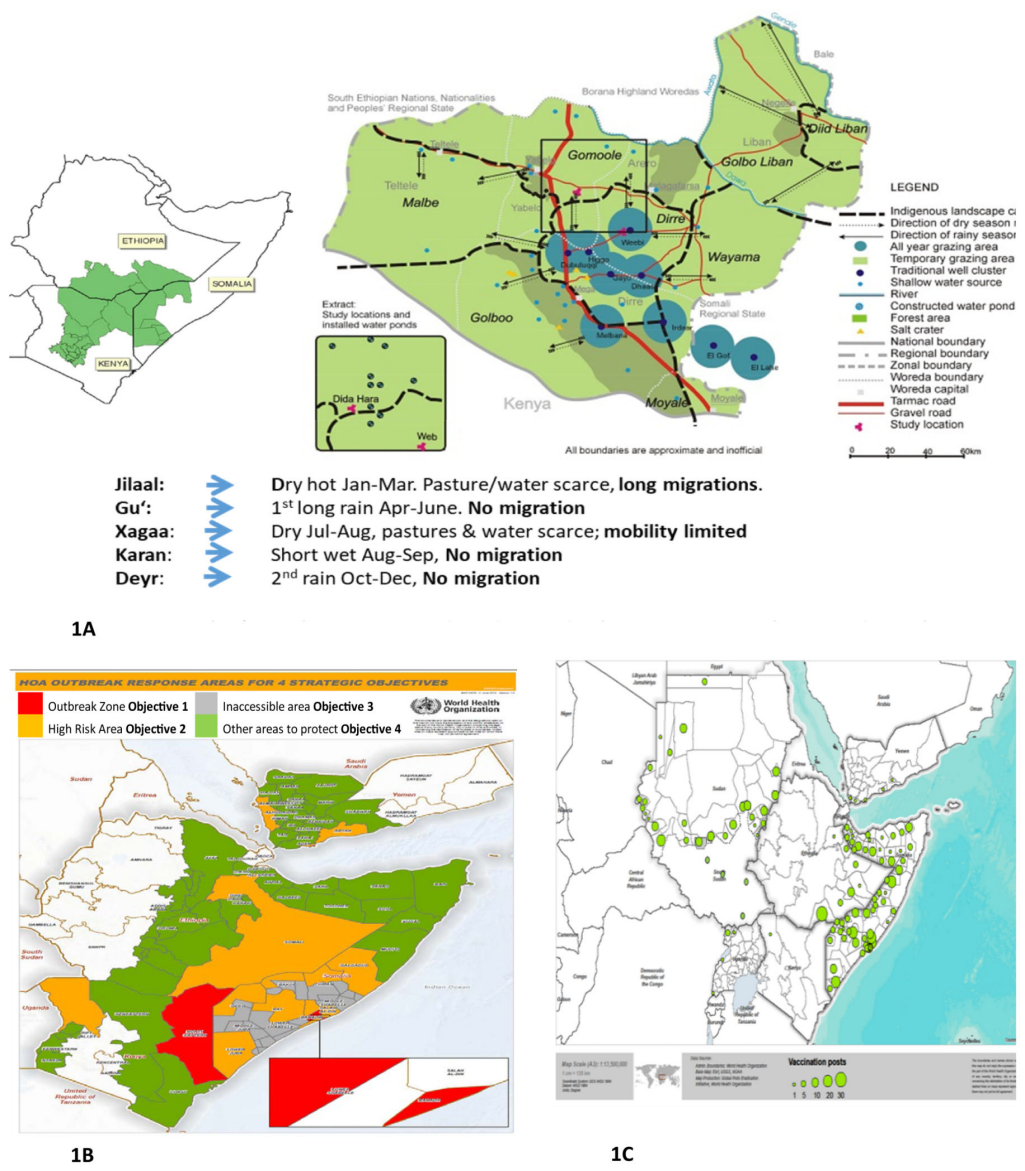
1A: Grazing path of Somali Borana Nomads and Nomadic clusters in Kenya, Ethiopia and Somalia 1B: HoA outreach response areas for four strategic objectives 1C: Map showing permamnent vaccination points (PVPs) in the HoA

More importantly, poliovirus outbreaks often transcend country, regional and geographical demarcations. With increasing globalization, population mobility is contributing to the transmission of infectious diseases between countries, as cross-border population movement impedes efforts to prevent transmission of infectious diseases^16^. For example, in 2009, 98.8% of total malaria cases in Yunnan Province, China were found to be imported from neighboring countries^16, 17^. In the HoA there is intense movement and interaction among the populations from the different countries across the two regions of AFRO and EMRO. Figure 1a shows the pathways of the Somalian pastoral/nomads traversing the whole length of Ethiopia, Kenya and Somalia. This group maintain close affinity with their kits and kin in Ethiopia and Kenya. It is thus not surprising that a poliovirus outbreak that debuts in Somalia is quickly linked to an outbreak in Kenya and Somali Region of Ethiopia.

The 2013 outbreak presented first in Somalia, then Kenya and later in Ethiopia. Given the links among these viruses, the outbreaks were considered as one. Though present in three different countries, the outbreak was in the same ethnic group with different clans the main ones being Jilaal, Gu, Xagaa, Karan and Deyr spread across three countries Ethiopia, Kenya, Somalia and the two WHO regions AFRO and EMRO (See Figure 1a). The outbreak areas in 2013 shown in red in Kenya and Somalia are surrounded by brown areas graded as high risk, the totally inaccessible areas shown in grey and finally the green areas considered low risk for transmission (Fig 1b). These risk zones cut across Kenya, Somalia and Ethiopia.

Characteristically, the area has high population movement which calls for coordinated supplementary immunization activities (SIAs) and other cross-cutting vaccination strategies, namely expanded age groups and short interval administered doses (SIAD) across borders.. In order to further increase chances of mobile populations and populations in hard to reach areas receiving immunization services, permanent vaccination points (PVP) were established along routes of mobile populations and in hard to reach areas in countries across the two WHO Regions as shown in fig 1c. Furthermore, other strategies namely, nomadic, SIA quality improvement, surveillance quality monitoring, AFP notification and laboratories called for cross regional efforts for efficient use of resources. The nomadic strategies entailed mapping of nomads, updating micro plans and engaging clan and traditional leaders from the start. Under this strategy also was the establishment of vaccination points at watering points. Improving SIAs called for expansion of immunization especially covering high risk areas as introduction and use of Lot Quality Assurance Sampling (LQAS) and the use of geographic positioning satellite and other hand held android phones.

Surveillance quality monitoring entailed the mapping of indicators and follow up of sub-optimal performances. Furthermore, is the need for smooth notification of AFP cases, addressing laboratory related issues, such as contact sampling. To alleviate workload, it became necessary to redistribution of stool samples, which called for the use of Uganda laboratory for samples from South Sudan for instance.

Beyond these were issues of communication, risk communication and social mobilization. These were the strong areas of expertise for UNICEF, a sister United Nations agency. Coordinating with UNICEF ESARO became expedient in engaging the communities and mobilizing them for behaviour change and acceptance of immunization at the different location. There were also other partner agencies like UNHCR, CDC, USAID, Red Cross, IOM, MMRS, and the CORE Group and local NGOs, all of which contributed immensely in responding to the outbreak in the HoA.

## Coordination Mechanism

The collaboration among these organizations and agencies across the different WHO regions and countries in the HoA, was facilitated with the establishment of the HoA coordination mechanism with office in Nairobi Kenya. The three Regional Directors of WHO AFRO and EMRO as well as the Regional Directors for UNICEF ESARO jointly communicated with the WHO and UNICEF countries representatives in the 10 HoA countries, intimating them of the HoA outbreak coordination office in Nairobi Kenya. Clear terms of reference were developed and operational funds were jointly mobilized and made available for response activities.

The establishment of the HoA cross-agency coordination group was another stimulus that drove the success of the response. Regular meetings were held in this group to appraise developments and develop joint plans to address emerging issues. These meetings were held daily in the early period of the outbreak, later weekly and much later fortnightly. Cross-border meetings were held at both national and local levels.

The coordination also used teleconferences with individual countries when necessary. There were also weekly teleconferences with the intercountry support teams (IST) Regional Offices and Headquarters. These in addition to outbreak calls with partners on a weekly basis. There were also regular visits to the three outbreak countries coordinated among the different agencies. Indeed, the coordination with WHO Regional Offices, Headquarters and UNICEF ESARO proved very useful. Budgets were submitted early and follow up of in-country transfer of funds to the operational levels were made seamless.

## Major Achievements

**Figure 2:**
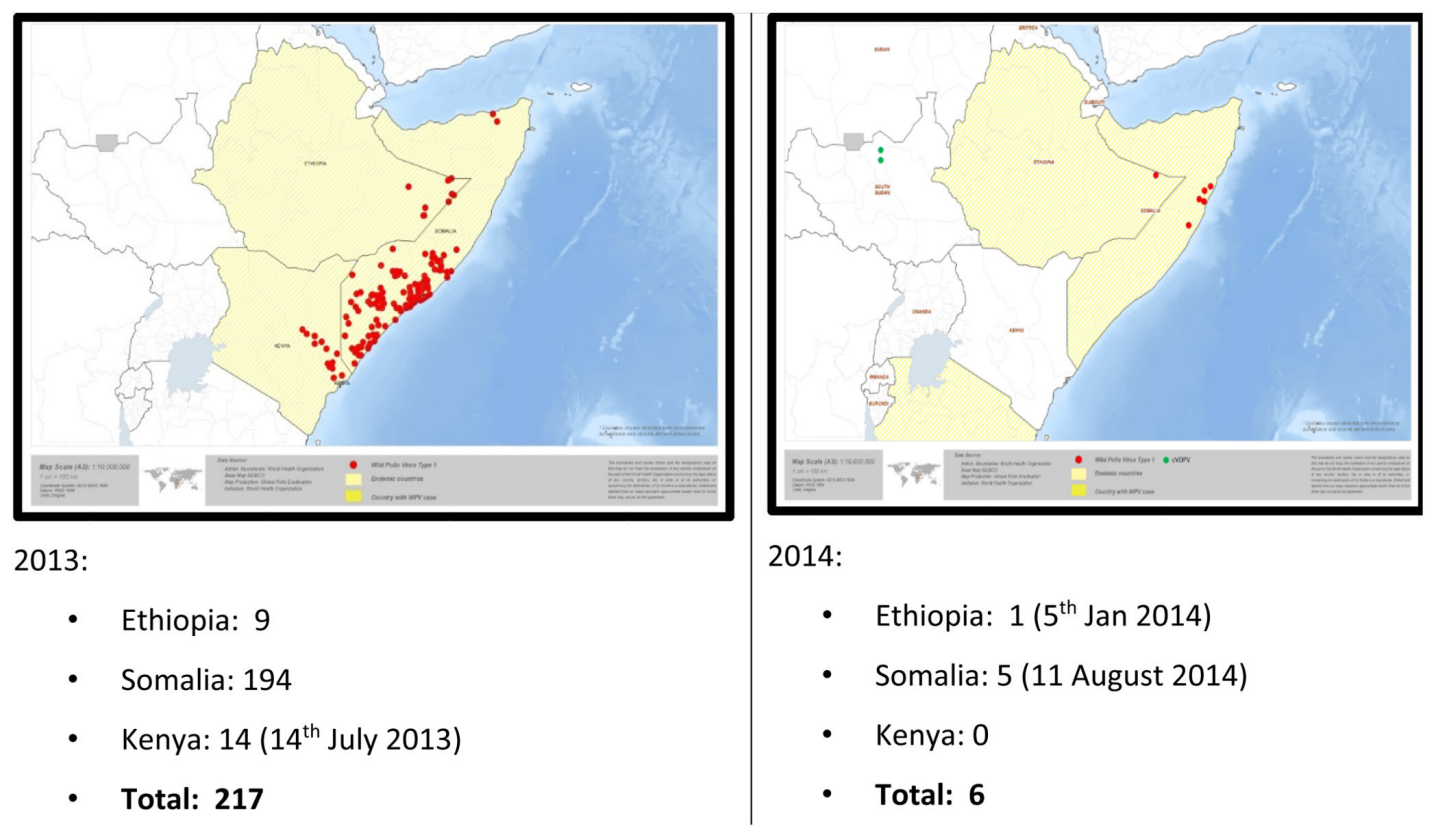
Sub regional maps of HoA Wild Polio Viruses in 2013 and 2014

The most critical achievement was that the wild polio virus (WPV) transmission in the HoA virtually stopped in a space of one year, despite the torrential waves in which they occurred. Figure 2 showed that between 2013 and 2014, the nine viruses in Ethiopia were reduced to one. In Somalia, within the same period, there was a drastic reduction from 194 WPVs to 11 WPVs by August 11, 2014. Similarly, in Kenya the 14 WPVs by July 14 2013 was reduced to zero in 2014. On the whole the total of 217 WPVs in the HoA in 2013 were brought down to six in 2014.

This remarkable success in the interruption of transmission of WPV in the HoA resulted from the coordinated implementation of the key GPEI strategies in the HoA sub regional. For instance, following each WPV, the response team conducted multiple SIAs in very short time. Table 1 revealed that in respect of the last outbreak of WPV in Somalia with date of onset 11 August 2014, there were four national immunization days (NIDs) two sub national immunization days (SNIDs) and three hard-to-reach short intervention administration doses (HTR SIADs). Similar actions were taken for the last WPV cases in Kenya with a date of onset 14 July 2013 and Ethiopia 5 January 2014.

**Figure 4:**
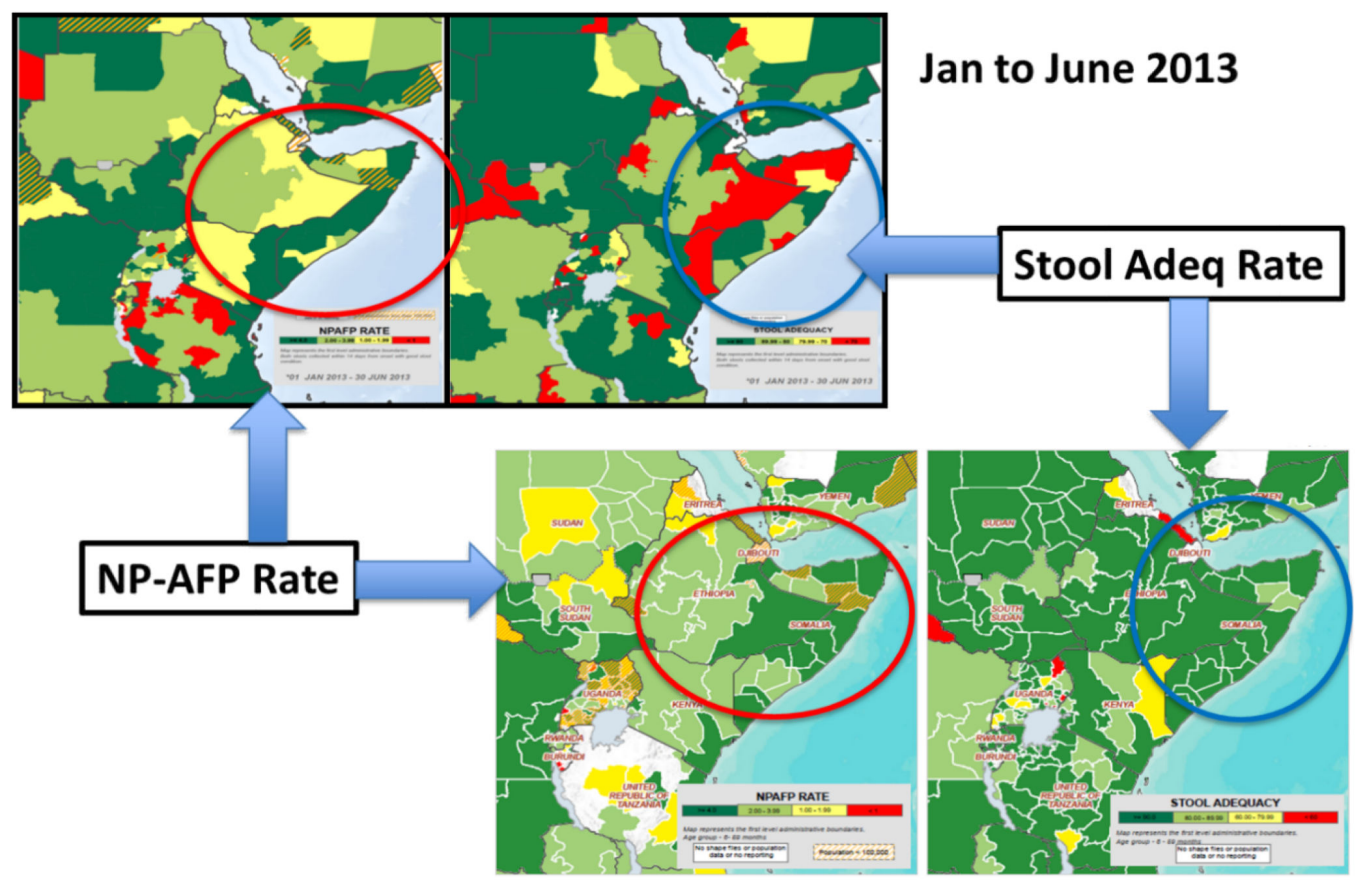
Improvements in the surveillance indicators

With the successful vaccination of the population came tremendous improvements in population immunity in the HoA. Figure 2 shows the steady reduction in the proportions of zero dose children <5years over the period of the outbreak response from 2013 to 2014. It showed a steady decline in areas where proportion of children who were zero dosed constituted ≥15 percent of the population of children aged <5 years. In addition to this, surveillance was intensified and made more sensitive leading to improvement in the two key surveillance indicators (See Figure 4). There was a progressive increase in the green portion of the maps showing the improvement in the stool adequacy and none polio AFP rate (NP-AFP). Reach to nomadic population also improved significantly. People who had never received vaccination services because of their geographic isolation were reached on the pastoral track through the fixed vaccination points.

Other major achievements include the establishment of permanent vaccination points, especially in Somalia where routine immunization is practically non-existent. It provides opportunities for children to get vaccination against poliovirus even after the response activities were closed. The response team also instituted the publication of many information platforms like the regular situation reports (SITREP) surveillance updates as well as weekly epidemiological updates and monthly bulletin. Two books were produced, namely *overview* of mobile populations in the Horn of Africa and Polio outbreak in the Horn of Africa: Best practices, lessons learned and innovations 2013-2014.

## Discussions and Lessons Learnt

The collaboration across the WHO region and among partner agencies facilitated the operations of the response team leading to the timeous interruption of poliovirus transmission in the HoA. This engendered timely information and resources sharing between the two WHO regions and the partner agencies. The critical importance of timely information sharing in arresting outbreaks cannot be over emphasized. The contribution of this element in outbreak response was document in the cross-border collaboration between China and Myanmar for emergency response to imported vaccine derived poliovirus case in the two countries with common geographical boundaries^17^. In that publication it was noted that timely information sharing between the two countries helped to eliminate potential outbreak of circulating vaccine derived poliovirus (cVDPV) in Myanmar, considering the immunity gap among children in this high risk area where the VDPV case lived.

The success in collaboration and the potential benefits attained emphasized the need to strengthen collaborative efforts among regions with neighbour-endemic countries. It is also critical that increasing efforts are committed to regional and cross-border collaboration in recent years. Studies have shown that intense cross-border migration continues in globally, favouring continuous virus transmission between countries^18^. It follows therefore that synchronized cross-border or cross regional polio campaigns were conducted in the two regions, ensuring simultaneous and comprehensive coverage of children in transit through the border areas to boost their immunity against polio viruses^19^. Furthermore, cross-border meetings have demonstrated the ability to help explore a formalized approach to collaboration^21, 22^.

The HoA polio outbreak response team promoted a health culture of cross regional participation under one coordination office facilitates the decision making processes which would otherwise be complicated if each Region was to take own decisions. Cross-Country Coordination of outbreak response activities under one office facilitates the synchronization and implementation of activities particularly along the international borders. Having the HOA Coordination enabled standardized approaches to monitoring and evaluation of the country activities summarized by each country on a weekly basis using a standardized template. The support of the Regional Offices AFRO, and EMRO, The WHO and UNICEF country Representatives was critical to facilitating the work of the Coordination Office especially linking with national authorities.

Another lesson learnt from the response was the effective use of the principle of delegation of authority. This was employed effectively in the response team. Delegation of authority to take decisions and actions at the coordination level was critical to ensure timely implementation of activities without reference to other levels which inherently leads to delays. The use of one Technical Advisory Group (TAG) across the Regions and for ten countries was instrumental in providing cross cutting recommendations which guided and supported implementation of activities across the Regions and countries. Having all relevant partners and actors under the same coordination office facilitated the decision making process across the partnership. Development of resource mobilization plan with one request to partners facilitated the mobilization of required resources.

The use of one Bulletin, One SITREP and one Donor Update prepared under the coordination office and shared with all the partners allowed same Technical and Management information to reach all concerned at the same time and reduced to a minimum additional requests for such information.

However, a number of challenges were encountered in the collaborative effort to respond to the polio outbreak in the HoA. First level of challenge was the financial flow. Despite all attempts to improve the flow of funds from the partners to countries and then to the operational ground, this remained a major challenge which sometimes led to postponement of SIA activities and as a result disorganize the cross border synchronization.

There was also a bit of lack of clarity among some partners on the overall coordination role invested in the HoA coordination office, in spite of the clear terms of reference issued the coordination office. When a policy decision was taken to relocate the WHO country Office for Somalia from Nairobi to Mogadishu, Communication and Coordination challenges ensued especially that the UNICEF Somalia Office remained in Nairobi. When this was realized, the WHO Polio Team leadership was relocated back to Nairobi and in the same office of UNICEF with very good results.

As the outbreak started to be controlled, competing priorities especially for partners agencies locally based in Nairobi started to take precedence with the result that the frequency of coordination meetings had to be curtailed to once every two weeks. This led to delays in taking some decisions as the coordination office had to do individual agency consultation to seek consensus in between meetings.

These challenges notwithstanding one can safely count the multiple benefits of the interregional and interagency collaboration. Beyond sharing of information and resources, it brought the comparative advantages of the different regions and partners on the table for common planning and simultaneous implementation of response activities. An uncoordinated immunity boosting in one area without the corresponding and timeous immunity boosting in the other would have been catastrophic and counterproductive. The transmission would have persisted if the improvements did not cut across the different regions simultaneously.

## Figures and Tables

**Figure 3:**
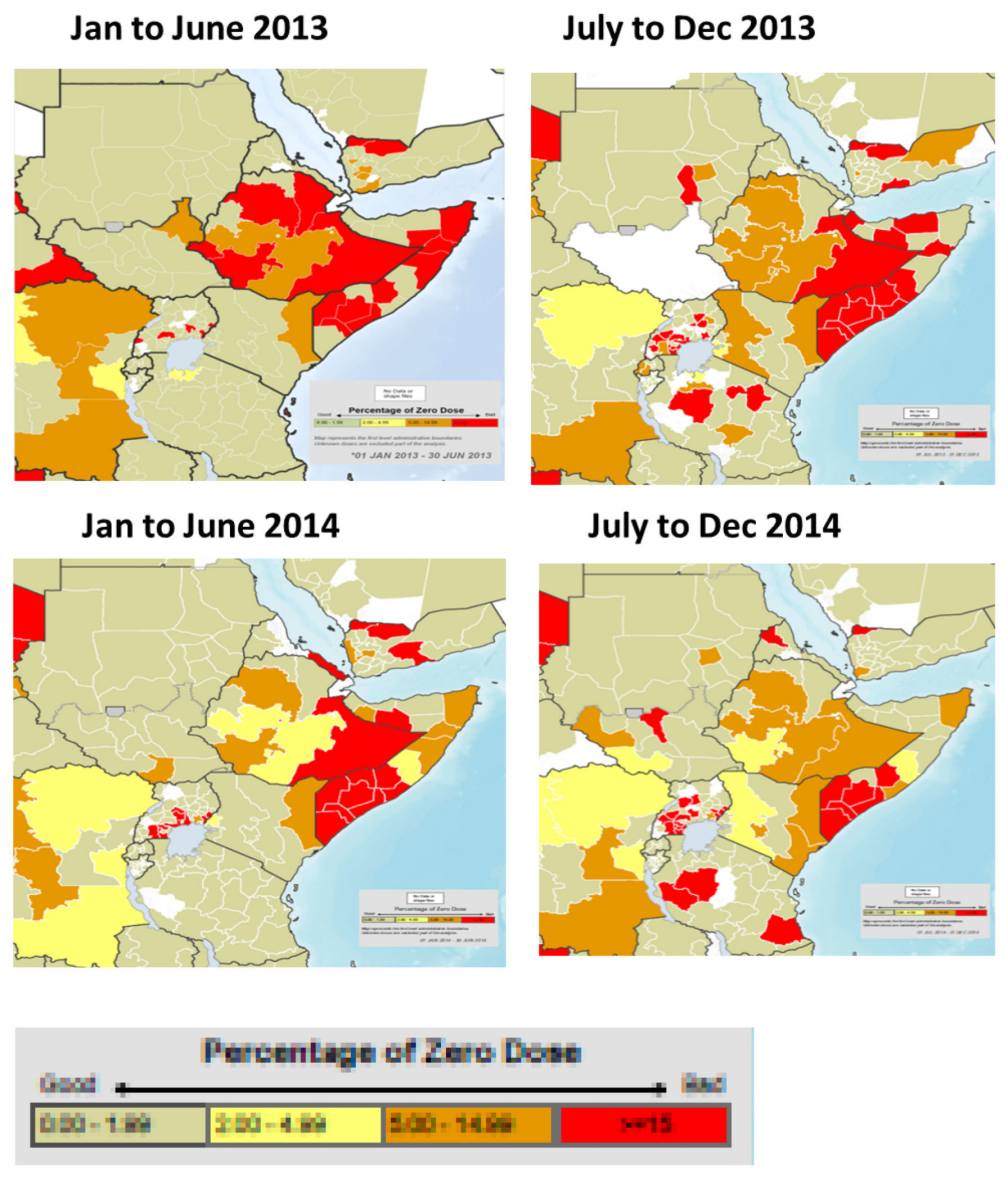
Improvements in population immunity

**Table 1:** SIAs conducted on response to the last WPV outbreaks in Somalia, Kenya and Ethiopia

Immunization Response	Somalia	Kenya	Ethiopia
Date of onset of last WPV	11^th^August 14	14^th^July 13	5^th^January 14
Number of SIAs in infected area after last WPV	4 NIDs	4 NIDs	
	2 SNIDs	9 SNIDs	2 NIDs
	3 HTR SIADs	1 Mop up	8 SNIDs
Number of SIAs since outbreak	15 NID		
	5 SNIDs	4 NIDs	4 NIDs
	3 HTR SIADs	14 SNIDs	13 SNIDs
	10 Mop ups	1 Mop up	2 Mop ups
Number of SIAs with tOPV since May 2013	1 Mop (2013)		
	1 SNID (2013)	1 NID (2013)	2 NID (2013, 2015)

NIDs – National immunization days

SNIDs – Sub-national immunization days

Mop-ups – Focused campaigns in areas with inadequate quality of the larger campaigns

SIADs – Short interval additional oral polio vaccine doses

HTR – Hard to reach areas
